# TRANSLATING SCIENCE INTO PRACTICE TO DEVELOP AN INNOVATIVE DEMENTIA CARE MODEL: THE HOMESTEAD CARE MODEL

**DOI:** 10.1093/geroni/igac059.1290

**Published:** 2022-12-20

**Authors:** Bram de Boer

**Affiliations:** Maastricht University, Maastricht, Limburg, Netherlands

## Abstract

This study reports on a participatory research approach to translate a scientific conceptual framework on innovative dementia care into practice. This led to the development of the Homestead care model. Results indicate that achieving positive outcomes for people with dementia, (in)formal caregivers, and the community is dependent on how well the physical, social and organizational environment are congruently designed. Physical aspects are related to interior design, outdoor areas, architecture and sensory elements. Social aspects include interactions with others in the environment, such as residents, staff, family, and the wider community (local entrepreneurs, societies, schools). Organizational aspects are related to the organizational culture and leadership of a care facility. These theoretical underpinnings of the conceptual model have been translated into three main pillars of the Homestead care model: activation, freedom, and relationships. The Homestead care model has a unique physical, social and organizational environment, which can affect daily life of residents.

